# Review on nanoparticles and nanostructured materials: history, sources, toxicity and regulations

**DOI:** 10.3762/bjnano.9.98

**Published:** 2018-04-03

**Authors:** Jaison Jeevanandam, Ahmed Barhoum, Yen S Chan, Alain Dufresne, Michael K Danquah

**Affiliations:** 1 Department of Chemical Engineering, Curtin University, CDT250 Miri, Sarawak 98009, Malaysia; 2 Department of Materials and Chemistry, Vrije Universiteit Brussel (VUB), Pleinlaan 2, 1050, Brussels, Belgium; 3 Chemistry Department, Faculty of Science, Helwan University, 11795 Helwan, Cairo, Egypt,; 4 University of Grenoble Alpes, CNRS, Grenoble INP, LGP2, F-38000 Grenoble, France

**Keywords:** nanomaterial classification, nanomaterial history, nanotoxicity, oxidative stress, reactive oxygen species, regulations

## Abstract

Nanomaterials (NMs) have gained prominence in technological advancements due to their tunable physical, chemical and biological properties with enhanced performance over their bulk counterparts. NMs are categorized depending on their size, composition, shape, and origin. The ability to predict the unique properties of NMs increases the value of each classification. Due to increased growth of production of NMs and their industrial applications, issues relating to toxicity are inevitable. The aim of this review is to compare synthetic (engineered) and naturally occurring nanoparticles (NPs) and nanostructured materials (NSMs) to identify their nanoscale properties and to define the specific knowledge gaps related to the risk assessment of NPs and NSMs in the environment. The review presents an overview of the history and classifications of NMs and gives an overview of the various sources of NPs and NSMs, from natural to synthetic, and their toxic effects towards mammalian cells and tissue. Additionally, the types of toxic reactions associated with NPs and NSMs and the regulations implemented by different countries to reduce the associated risks are also discussed.

## Review

### Introduction

Nanoparticles (NPs) and nanostructured materials (NSMs) represent an active area of research and a techno-economic sector with full expansion in many application domains. NPs and NSMs have gained prominence in technological advancements due to their tunable physicochemical characteristics such as melting point, wettability, electrical and thermal conductivity, catalytic activity, light absorption and scattering resulting in enhanced performance over their bulk counterparts. A nanometer (nm) is an International System of Units (Système international d'unités, SI) unit that represents 10^−9^ meter in length. In principle, NMs are described as materials with length of 1–1000 nm in at least one dimension; however, they are commonly defined to be of diameter in the range of 1 to 100 nm. Today, there are several pieces of legislation in the European Union (EU) and USA with specific references to NMs. However, a single internationally accepted definition for NMs does not exist. Different organizations have a difference in opinion in defining NMs [1]. According to the Environmental Protection Agency (EPA), “NMs can exhibit unique properties dissimilar than the equivalent chemical compound in a larger dimension” [2]. The US Food and Drug Administration (USFDA) also refers to NMs as “materials that have at least one dimension in the range of approximately 1 to 100 nm and exhibit dimension-dependent phenomena” [3]. Similarly, The International Organization for Standardization (ISO) has described NMs as a “material with any external nanoscale dimension or having internal nanoscale surface structure” [4]. Nanofibers, nanoplates, nanowires, quantum dots and other related terms have been defined based on this ISO definition [5]. Likewise, the term *nanomaterial* is described as “a manufactured or natural material that possesses unbound, aggregated or agglomerated particles where external dimensions are between 1–100 nm size range”, according to the EU Commission [6]. Recently, the British Standards Institution [7] proposed the following definitions for the scientific terms that have been used:

- Nanoscale: Approximately 1 to 1000 nm size range.
- Nanoscience: The science and study of matter at the nanoscale that deals with understanding their size and structure-dependent properties and compares the emergence of individual atoms or molecules or bulk material related differences.
- Nanotechnology: Manipulation and control of matter on the nanoscale dimension by using scientific knowledge of various industrial and biomedical applications.
- Nanomaterial: Material with any internal or external structures on the nanoscale dimension.
- Nano-object: Material that possesses one or more peripheral nanoscale dimensions.
- Nanoparticle: Nano-object with three external nanoscale dimensions. The terms nanorod or nanoplate are employed, instead of nanoparticle (NP) when the longest and the shortest axes lengths of a nano-object are different.
- Nanofiber: When two similar exterior nanoscale dimensions and a third larger dimension are present in a nanomaterial, it is referred to as nanofiber.
- Nanocomposite: Multiphase structure with at least one phase on the nanoscale dimension.
- Nanostructure: Composition of interconnected constituent parts in the nanoscale region.
- Nanostructured materials: Materials containing internal or surface nanostructure.

The use of various definitions across different jurisdictions acts as a major hurdle to regulatory efforts as it leads to legal hesitation in applying regulatory approaches for identical NMs. Therefore, the need to satisfy diverging considerations is a major challenge in developing a single international definition for NMs.

### Types and classification of nanomaterials

Most current NPs and NSMs can be organized into four material-based categories (the references refer to recent reviews on these different categories of NMs).

**Figure 1:**
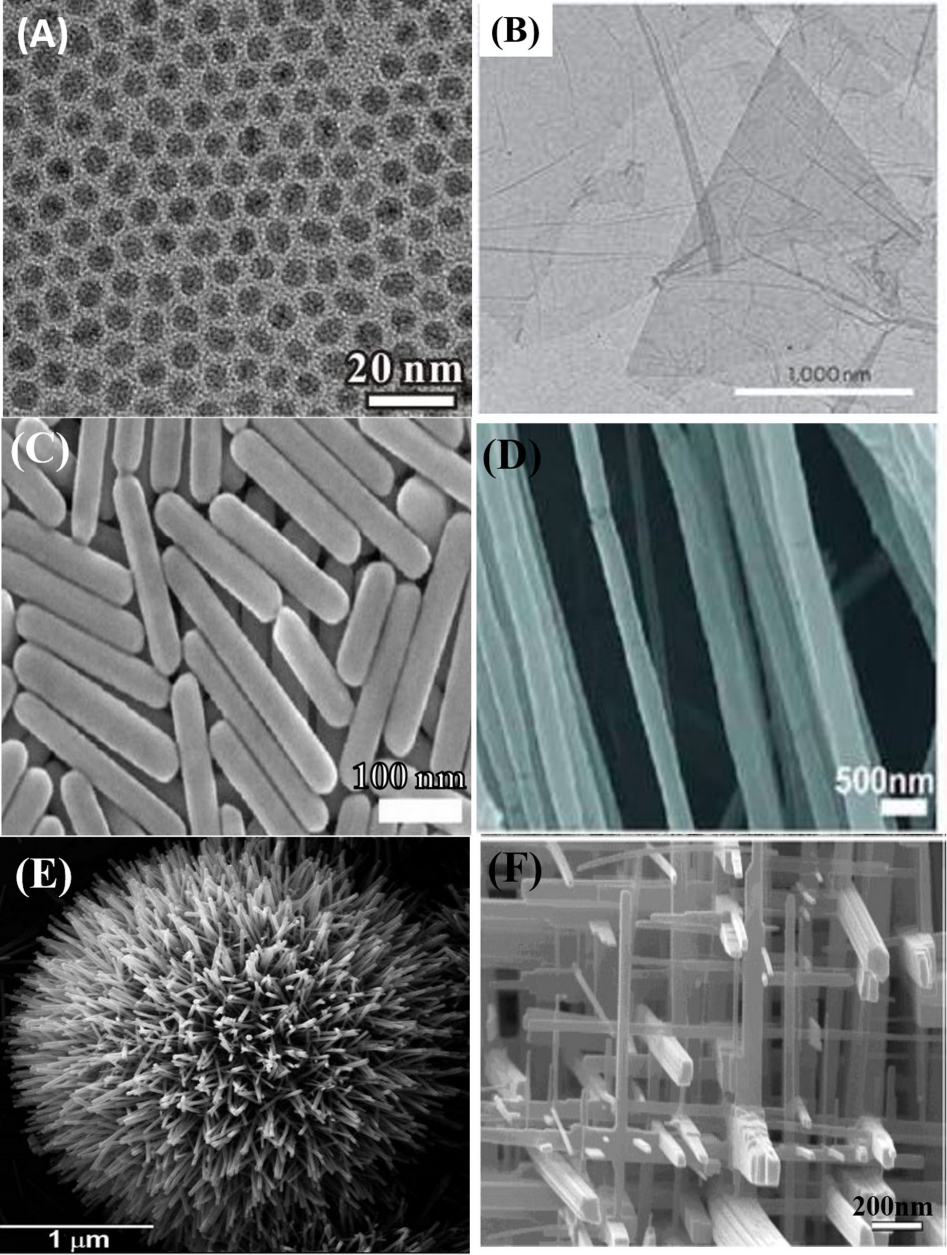
Nanomaterials with different morphologies: (A) nonporous Pd NPs (0D) [9–10], copyright Zhang et al.; licensee Springer, 2012, (B) Graphene nanosheets (2D) [11], copyright 2012, Springer Nature, (C) Ag nanorods (1D) [12], copyright 2011, American Chemical Society, (D) polyethylene oxide nanofibers (1D) [13], copyright 2010, American Chemical Society, (E) urchin-like ZnO nanowires (3D), reproduced from [14] with permission from The Royal Society of Chemistry, (F) WO_3_ nanowire network (3D) [15], copyright 2005 Wiley-VCH.

(i) Carbon-based nanomaterials: Generally, these NMs contain carbon, and are found in morphologies such as hollow tubes, ellipsoids or spheres. Fullerenes (C60), carbon nanotubes (CNTs), carbon nanofibers, carbon black, graphene (Gr), and carbon onions are included under the carbon-based NMs category. Laser ablation, arc discharge, and chemical vapor deposition (CVD) are the important production methods for these carbon-based materials fabrication (except carbon black) [8].

(ii) Inorganic-based nanomaterials: These NMs include metal and metal oxide NPs and NSMs. These NMs can be synthesized into metals such as Au or Ag NPs, metal oxides such as TiO_2_ and ZnO NPs, and semiconductors such as silicon and ceramics.

(iii) Organic-based nanomaterials: These include NMs made mostly from organic matter, excluding carbon-based or inorganic-based NMs. The utilization of noncovalent (weak) interactions for the self-assembly and design of molecules helps to transform the organic NMs into desired structures such as dendrimers, micelles, liposomes and polymer NPs.

(iv) Composite-based nanomaterials: Composite NMs are multiphase NPs and NSMs with one phase on the nanoscale dimension that can either combine NPs with other NPs or NPs combined with larger or with bulk-type materials (e.g., hybrid nanofibers) or more complicated structures, such as a metal-organic frameworks. The composites may be any combinations of carbon-based, metal-based, or organic-based NMs with any form of metal, ceramic, or polymer bulk materials. NMs are synthesized in different morphologies as mentioned in Figure 1 depending on the required properties for the desired application.

#### Classification of nanomaterials based on their dimensions

The production of conventional products at the nanoscale currently helps and will continue to will help the economic progress of numerous countries. Many types of NPs and NSMs have been reported and many other varieties are predicted to appear in the future. Therefore, the need for their classification has ripened. The first idea for NM classification was given by Gleiter et al. [16]. Here, NMs were classified depending on their crystalline forms and chemical composition. However, the Gleiter scheme was not fully complete because the dimensionality of the NPs and NSMs was not considered [17]. In 2007, Pokropivny and Skorokhod made a new scheme of classification for NMs which included the recently developed composites such as 0D, 1D, 2D and 3D NMs, as shown in Figure 1 [18]. This classification is highly dependent on the electron movement along the dimensions in the NMs. For example, electrons in 0D NMs are entrapped in a dimensionless space whereas as 1D NMs have electrons that can move along the *x*-axis, which is less than 100 nm. Likewise, 2D and 3D NMs have electron movement along the *x*–*y*-axis, and *x*, *y*, *z*-axis respectively.

The ability to predict the properties of NMs determines the classification value of the NMs. The properties of NMs strongly depend on the grain boundaries, as mentioned in the “grain boundary engineering” concept in Gleiter's classification. Therefore, the classical inner size effects, such as melting point reduction and diffusion enhancement, will be enhanced by grain boundary engineering. The classification by Pokropivny and Skorokhod proposed that the characteristics of NMs are attributed to the particle shape and dimensionality, as per the “surface engineering” concept, and thereby class of NMs. Thus, these reasons focus on the engineering of particle shape and dimensionality along with grain boundary engineering to extend the application of NSMs [18].

#### Classification of nanomaterials based on their origin

Apart from dimension and material-based classifications, NPs and NSMs can also be classified as natural or synthetic, based on their origin.

(i) Natural nanomaterials are produced in nature either by biological species or through anthropogenic activities. The production of artificial surfaces with exclusive micro and nanoscale templates and properties for technological applications are readily available from natural sources. Naturally occurring NMs are present throught the Earth’s spheres (i.e., in the hydrosphere, atmosphere, lithosphere and even in the biosphere), regardless of human actions. Earth is comprised of NMs that are naturally formed and are present in the Earth’s spheres, such as the atmosphere, which includes the whole of troposphere, the hydrosphere, which includes oceans, lakes, rivers, groundwater and hydrothermal vents, the lithosphere, which is comprised of rocks, soils, magma or lava at particular stages of evolution and the biosphere, which covers micro-organisms and higher organisms, including humans [19–20].

(ii) Synthetic (engineered) nanomaterials are produced by mechanical grinding, engine exhaust and smoke, or are synthesized by physical, chemical, biological or hybrid methods. The question of risk assessment strategies has arisen in recent times as there is increased fabrication and subsequent release of engineered NMs as well as their usage in consumer products and industrial applications. These risk assessment strategies are highly helpful in forecasting the behavior and fate of engineered NMs in various environmental media. The major challenge among engineered NMs is whether existing knowledge is enough to forecast their behavior or if they exhibit a distinct environment related behavior, different from natural NMs. Currently, different sources related to potential applications are used for the production of engineered NMs [21].

### History and development of nanomaterials

Humans already exploited the reinforcement of ceramic matrixes by including natural asbestos nanofibers more than 4,500 years ago [22]. The Ancient Egyptians were also using NMs more than 4000 years ago based on a synthetic chemical process to synthesize ≈5 nm diameter PbS NPs for hair dye [23]. Similarly, “Egyptian blue” was the first synthetic pigment which was prepared and used by Egyptians using a sintered mixture nanometer-sized glass and quartz around 3rd century BC [24]. Egyptian blue represents a multifaceted mixture of CaCuSi_4_O_10_ and SiO_2_ (both glass and quartz). In ancient geographical regions of the Roman Empire, including countries such as Egypt, Mesopotamia, and Greece, the extensive use of Egyptian blue for decorative purposes has been observed during archaeological explorations.

The synthesis of metallic NPs via chemical methods dates back to the 14th and 13th century BC when Egyptians and Mesopotamians started making glass using metals, which can be cited as the beginning of the metallic nanoparticle era [25]. These materials may be the earliest examples of synthetic NMs in a practical application. From the late Bronze Age (1200–1000 BC), red glass has been found in Frattesina di Rovigo (Italy) that is colored by surface plasmon excitation of Cu NPs [26]. Similarly, the Celtic red enamels originating from the 400–100 BC period have been reported to contain Cu NPs and cuprous oxide (cuprite Cu_2_O) [27]. Nevertheless, a Roman glass workpiece is the most famous example of ancient metallic NPs usage. The Lycurgus Cups are a 4th-century Roman glass cup, made of a dichroic glass that displays different colors: red when a light passes from behind, and green when a light passes from the front [28]. Recent studies found that the Lycurgus Cups contain Ag–Au alloy NPs, with a ratio of 7:3 in addition to about 10% Cu [29]. Later, red and yellow colored stained glass found in medieval period churches was produced by incorporating colloidal Au and Ag NPs, respectively [25]. During the 9th century, Mesopotamians started using glazed ceramics for metallic luster decorations [22]. These decorations showed amazing optical properties due to the existence of distinct Ag and/or Cu NPs isolated within the outermost glaze layers. These decorations are an example of metal nanoparticles that display iridescent bright green and blue colors under particular reflection conditions. TEM analysis of these ceramics revealed a double layer of Ag NPs (5–10 nm) in the outer layer and larger ones (5–20 nm) in the inner layer. The distance was observed to be constant at about 430 nm in between two layers, giving rise to interference effects. The scattered light from the second layer leads to the phase shift due to the scattering of light by the first layer. This incoming light wavelength dependent phase shift leads to a different wavelength while scattering. Later, the red glass was manufactured using this process all over the world. In the mid-19th century, a similar technique was used to produce the famous Satsuma glass in Japan. The absorption properties of Cu NPs were helpful in brightening the Satsuma glass with ruby color [30]. Furthermore, clay minerals with a thickness of a few nanometers are the best examples of natural NM usage since antiquity. It was reported that even in 5000 BC, clay was used to bleach wools and clothes in Cyprus [31].

In 1857, Michael Faraday reported the synthesis of a colloidal Au NP solution, which is the first scientific description to report NP preparation and initiated the history of NMs in the scientific arena. He also revealed that the optical characteristics of Au colloids are dissimilar compared to their respective bulk counterpart. This was probably one of the earlier reports where quantum size effects were observed and described. Later, Mie (1908) explained the reason behind the specific colors of metal colloids [32]. In the 1940s, SiO_2_ NPs were being manufactured as substitutes to carbon black for rubber reinforcement [33]. Today manufactured NMs can significantly improve the characteristics of bulk materials, in terms of strength, conductivity, durability, and lightness, and they can provide useful properties (e.g., self-healing, self-cleaning, anti-freezing, and antibacterial) and can function as reinforcing materials for construction or sensing components for safety. Notwithstanding the other possible benefits, simply taking advantage of the beneficial size and shape effects to improve the appearance of materials is still a major application of NPs. Moreover, the commercial use of NMs is often limited to the bulk use of passive NMs embedded in an inert (polymer or cement) matrix, forming a nanocomposite. In 2003, Samsung introduced an antibacterial technology with the trade name Silver Nano™ in their washing machines, air conditioners, refrigerators, air purifiers and vacuum cleaners, which use ionic Ag NPs [34]. NPs and NSMs are extensively used in auto production: as fillers in tires to improve adhesion to the road, fillers in the car body to improve the stiffness, and as transparent layers used for heated, mist and ice-free, window panes [35]. By the end of 2003, Mercedes-Benz brought a NP-based clear coat into series production for both metallic and nonmetallic paint finishes. The coating increases the scratch resistance and enhances the gloss. Liquid magnets, so-called ferrofluids, are ultrastable suspensions of small magnetic NPs with superparamagnetic properties [36]. Upon applying a magnetic field, the liquid will macroscopically magnetize, which leads to the alignment of NPs along the magnetic field direction [37]. Recent research has focused on creating enhanced Earth-based astronomical telescopes with adaptive optics and magnetic mirrors with the shape-shifting capability made up of ferrofluids [38–39]. TiO_2_ NPs are commercially used in solar cells with dye-sensitization ability [40]. In summer 2012, Logitech brought an external iPad keyboard powered by light on the market, representing the first major commercial use of dye-sensitized solar cells. In 2005, Abraxane™, which is a human serum albumin NP material containing paclitaxel, was manufactured, commercialized and released in the pharmaceutical market [41]. In 2014, there were about 1814 nanotechnology-based consumer products that are commercially available in over 20 countries [42].

### Sources of nanomaterials

Sources of nanomaterials can be classified into three main categories based on their origin: (i) incidental nanomaterials, which are produced incidentally as a byproduct of industrial processes such as nanoparticles produced from vehicle engine exhaust, welding fumes, combustion processes and even some natural process such as forest fires; (ii) engineered nanomaterials, which have been manufactured by humans to have certain required properties for desired applications and (iii) naturally produced nanomaterials, which can be found in the bodies of organisms, insects, plants, animals and human bodies. However, the distinctions between naturally occurring, incidental, and manufactured NPs are often blurred. In some cases, for example, incidental NMs can be considered as a subcategory of natural NMs.

Molecules are made up of atoms, which are the basic structural components of all living and nonliving organisms in nature. Atoms and molecules have been naturally manipulated several times to create intricate NPs and NSMs that continually contribute to life on earth. Incidental and naturally occurring NMs are continuously being formed within and distributed throughout ground and surface water, the oceans, continental soil, and the atmosphere. One of the main differences between incidental and engineered NMs is that the morphology of engineered NMs can usually be better controlled as compared to incidental NMs; additionally, engineered NMs can be purposely designed to exploit novel features that stem from their small size. It is known that metal NPs may be spontaneously generated from synthetic objects, which implies that humans have long been in direct contact with synthetic NMs and that macroscale objects are also a potential source of incidental nanoparticles in the environment.

#### Incidental nanomaterials

Photochemical reactions, volcanic eruptions, and forest fires are some of the natural processes that lead to the production of natural NPs as mentioned. In addition, skin and hair shedding of plants and animals, which is frequent in nature, contributes to NP composition in nature. Dust storms, volcanic eruptions, and forest fires are events of natural origin that are reported to produce high quantities of nanoparticulate matter that significantly affect worldwide air quality. Similarly, transportation, industrial operations, and charcoal burning are some of the human activities that lead to the emergence of synthetic NPs. Only about 10% of overall aerosols in the atmosphere are generated by human activity, whereas the naturally generated ones amount to 90% of atmospheric aerosols [43].

**Dust storms and cosmic dust:** The Eagle Nebula stars are 6500 light years away from Earth and are born with a disk-like cloud and the ability to form solar systems accompanied by dust and gas (mostly hydrogen) [44]. Astronomical observations (especially infrared spectroscopy) and direct “stardust” analysis during space missions and meteorite collections determined that the vast assortment of carbide, oxide, nitride, silicate, carbon, and organic-based NMs are the main components of stardust [44]. Diamond, of a few nanometers in diameter, has been observed in the Murchison meteorite, which is a perfect example of the nanoparticulate origin in planetary system objects other than stars [45]. Different types of NMs are present throughout the universe which are mixed, sorted and modified into several forms. Electromagnetic radiation, pressure gradients, dramatic temperature, physical collisions and shock waves help in energizing and forming NPs in space [44]. This leads to the widest range of nanoscale materials with distinct re-equilibration/phase mixing and isomerization along the chemical spectrum [19].

Dust storms are the main source of NPs in desert and terrestrial regions. Studies supported by satellite images revealed that dust storms in one region can migrate the nano and micro-sized minerals and anthropogenic pollutants to thousands of kilometers away from their origin. About 50% of the atmospheric aerosol particles that originate from dust storms in deserts are in the range of 100–200 nm [46–47]. The consequence of aerosol particles on the environment and climate was extensively reviewed by Buseck and Posfai. They mentioned that widespread transport of aerosols across oceans have a major effect on life, including the life forms at the bottom of the food chain [48]. Another study by Al-Dabbous and Prashant Kumar revealed the presence of 5–1000 nm range airborne NPs during summertime and dust events in busy roadsides (terrestrial) of Kuwait [49].

**Figure 2:**
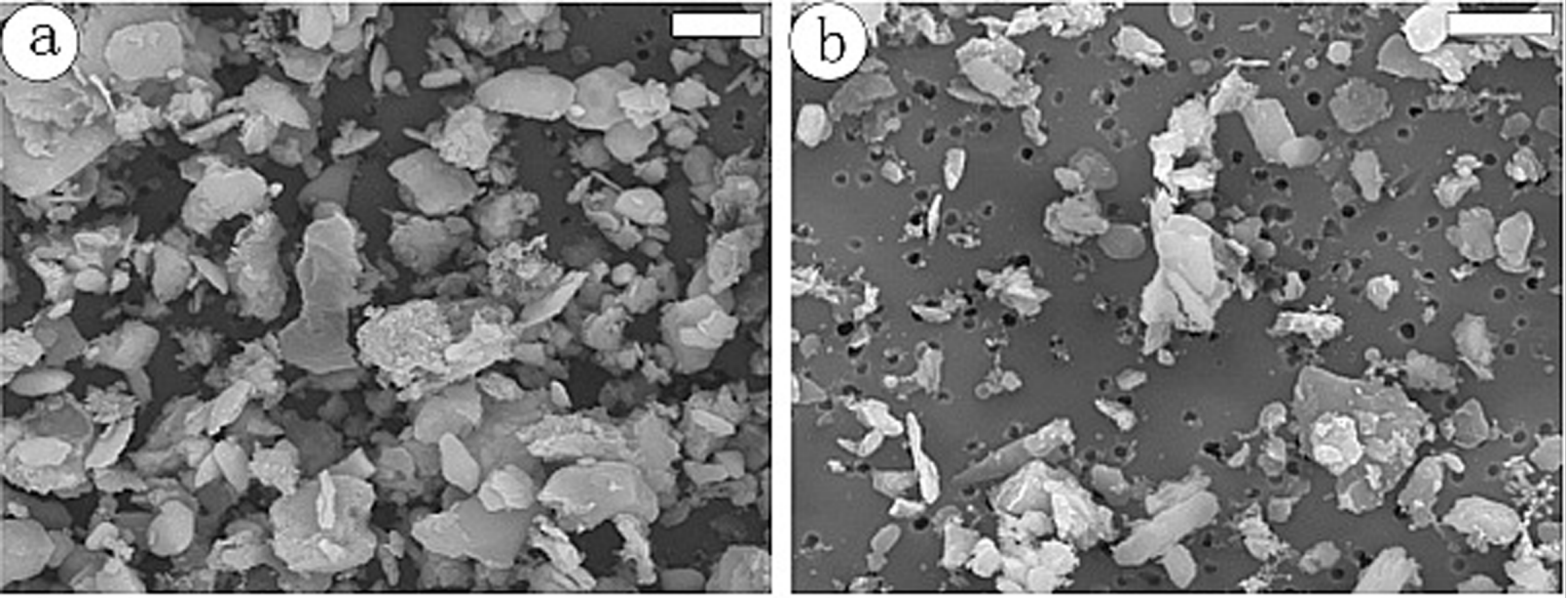
FESEM of dust particle samples collected (a) during and (b) after the dust storm episodes on March 16, 2002 (scale bar 5 μm) [47], copyright 2005, the American Geophysical Union.

Asthma and emphysema are two prominent health problems in humans that are caused by terrestrial airborne dust particles [50–51]. Dust NPs containing metals have the capability of damaging lung tissues by producing reactive oxygen species [43]. A case study shows that the quality of air in Asia and North America is heavily disturbed during every spring season due to dust storms occurring in the Gobi desert [52–53]. More recently, Shi et al. (2009) also reported (through simulated cloud processing) that dust storms help to form Fe NPs in clouds, which creates pH fluctuations, and affects the atmospheric, mineralogical, physical and chemical properties of the Saharan desert region [54–57]. Figure 2 is an example of aggregated NPs present in a dust storm region during and after dust storms.

Cosmic dust is a collection of extraterrestrial dust particles that widely exist in space on the nanoscale. Many meteorites and extraterrestrial materials have been found to possess natural NMs, which were extensively listed in the review “Nanotechnology: nature’s gift or scientists’ brainchild?” [19]. The astronauts and aeronautic instruments are severely threatened by cosmic dust [58]. Lunar dust is smaller compared to typical terrestrial dust, with excess sub-micrometer particles. Lunar dust, with a few magnetic NPs, can settle on the space suits of astronauts by electrostatic attraction and damage them [59–60]. They have been known to cause irritation in the lungs and eyes of Apollo astronauts by becoming airborne [61]. Studies have found that through the intratracheal route, lunar materials lead to pneumoconiosis and fibrosis formation in rats [62]. Dust particles on Mars can damage the solar panels of the exploration robots via accumulation and affects the power source for sensing, communication, and locomotion [63]. Astronauts who are frequently on longer space missions have prolonged exposure to cosmic dust with an increased risk of respiratory disease. Dust particles also cause damage and mechanical failure in spacesuits and airlocks [64].

**Volcanic eruptions:** Eruption of volcanoes leads to the propulsion of an enormous amount of aerosols and fine particles into the atmosphere with sizes ranging from micrometers to several nanometers [64–67]. A single volcanic eruption can release up to 30 × 10^6^ tons of NPs in the form of ash into the atmosphere [43]. The released NPs spread throughout the world and settle in the stratosphere and the troposphere, which are the lowest atmospheric layers. However, the effect of NPs will be significant in areas within a certain range (10 km) from the volcano. Rietmeijer and Mackinnon reported that volcanic eruptions in the 1980s resulted in the release of bismuth oxide NPs into the stratosphere and were detected even in1985 [68]. Particulate debris from volcanic eruptions affects human, animal, and plant activities by blocking and scattering the sunlight. The volcanically erupted particles may possess heavy metals that are toxic to humans [69]. The short-term effects of particles from volcanic eruptions include nose, throat, eye and skin irritations and bronchial symptoms, while the long-term effects include diseases such as podocinids [70–72] and Kaposi’s sarcoma [73–74]. Podoconiosis is caused by the micro- or nanoparticle absorption from the soil through the feet’s skin, leading to localized fluid retention in the lower limbs [75]. Kaposi’s sarcoma is similar to cancer and human herpes virus infection that affects the blood and lymph vessels. It is caused by the entry of NPs into the body [73].

**Figure 3:**
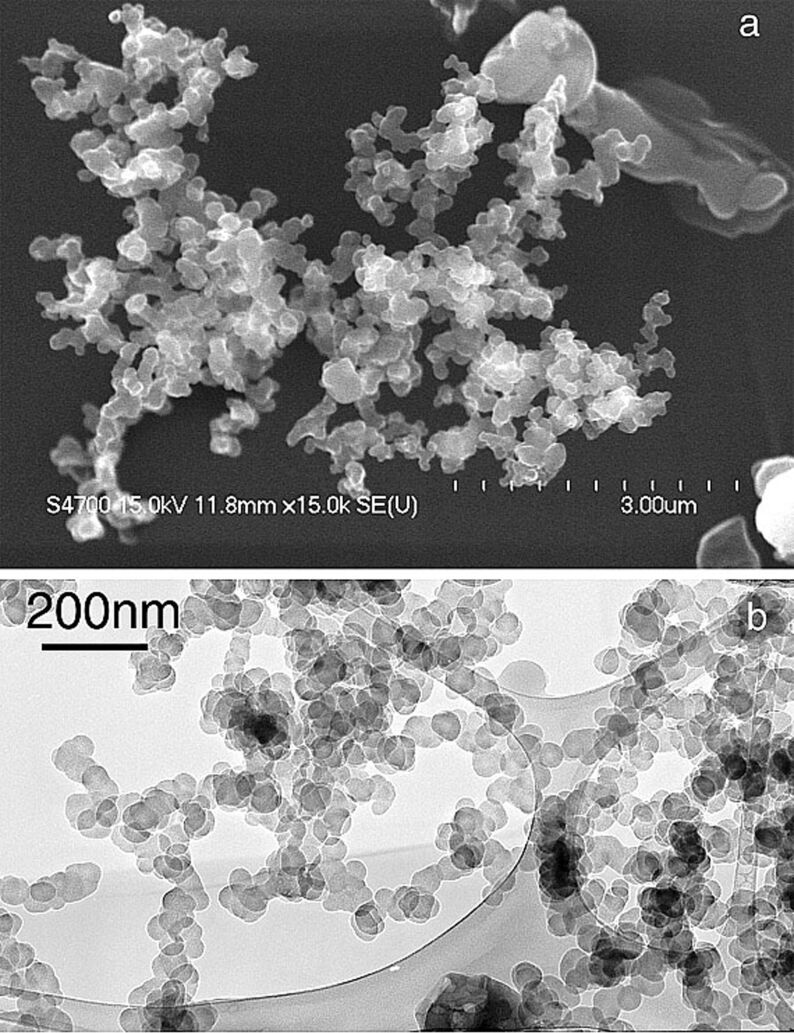
(a) SEM image of flaming smoke collected during a Madikwe Game Reserve fire in South Africa on August 20, 2000, showing aggregated carbon particles; (b) TEM image of flaming smoke collected in a Dambo fire in Zambia, on September 5, 2000, showing aggregated carbon particles [82], copyright 2003, the American Geophysical Union.

**Forest fires and ocean water evaporation:** Lightning and human activity are the main causes of forest and grass fires across the world. Ash and smoke are released by these forest fires and can spread over long distances, affecting the standard of ambient air quality by increasing the number of small particles in the air [50]. It has been shown that that black carbon and soot in large quantities are carried and deposited over the Himalayan glaciers by Asian brown clouds. These deposited particles are the primary reason for increased absorption of the sun’s heat and accelerate the glacial melting process [76–77]. Figure 3 is an example of nanoparticulates present in the smoke. Many forest fire cases have been reported to transport micro- and nanosized particles through smoke and ash, and are known to cause respiratory problems in humans and animals [78–80]. Smoke containing very small particles can worsen pre-existing cardiopulmonary conditions in patients [73]. It has also been reported that smoke inhalation causes 75% of fire-related deaths [64]. Sea salt aerosols are a different type of natural NPs formed due to water evaporation and ejection of wave-produced water droplets from seas and oceans into the atmosphere [48]. Usually, the size of these salt aerosols ranges from 100 nm to few micrometers, and are formed via temperature change and evaporation-mediated natural precipitation. It has been reported that formation of CaCO_3_ NPs in Lake Michigan is due to weather and temperature changes [81]. These small sea salt aerosols act to transfer microorganisms and pollutants that may increase casualties in plants, animals, and humans via adverse health effects.

#### Engineered nanomaterials

Simple combustion during cooking, in vehicles, fuel oil and coal for power generation [83], airplane engines, chemical manufacturing, welding, ore refining and smelting are some of the anthropogenic activities that lead to NP formation [84]. NMs such as carbon NPs [85], TiO_2_ NPs [86] and hydroxyapatites [87] are present in commercial cosmetics, sporting goods, sunscreen and toothpaste. Thus, these synthetic NPs are a new genre of NPs that may induce adverse environmental and human health effects.

**Nanoparticles from diesel and engine exhaust:** In cosmopolitan cities and town, the main source of atmospheric micro- and nanoparticles is automobile exhaust [88]. Amongst the types of automobile exhaust, diesel engines release 20–130 nm sized particles whereas gasoline engines release 20–60 nm sized particles [89–90]. It has been found that CNTs and fibers are released as by-products during diesel and gas combustion processes [91]. More than 90% of carbon NPs present in the atmosphere are diesel-generated particles [92]. Thus, pollution from vehicles is a major cause of nanoparticulate contamination in urban atmosphere [93]. The hazardous effect of automobile exhaust depends on the composition of the particulate mixture [94]. Recently, fine particulate matter, especially carbon nanotubes of anthropogenic origin, was found to be present in the broncho-alveolar lavage fluids from asthmatic Parisian children. The results showed that the presence of carbon nanotubes in cells can cause granulomatous reactions, oxidative stress and inflammation, leading to fibroplasia and neoplasia in lungs. The results also suggested that humans are routinely exposed to carbon nanotubes and showed that the outcome is similar to the vehicle exhaust samples collected in Paris, ambient air samples from the USA, a spider web sample in India and in ice core [95]. Also, benzo[*a*]pyrene, which is a polynuclear aromatic hydrocarbon and a carcinogen, is present in diesel exhaust, which makes it more toxic than gas engine exhaust [96]. Cardiopulmonary mortality [97–98], childhood cancers due to prenatal and postnatal exposure to exhaust [99], myocardial infarction [100], and proinflammatory, prothrombotic and hemolytic responses [101] are some of the health problems that are observed in humans due to high exposure to exhaust in highly populated cities.

**Cigarette smoke and building demolition:** Cigarette smoking and building demolition are anthropogenic activities that lead to the spread of NPs into the atmosphere. Cigarette smoke has a complex composition of about 100,000 chemical compounds in the form of NPs ranging from 10–700 nm [102]. Similarly, nano- and microparticulates smaller than 10 μm are released into the atmosphere when larger buildings are demolished [103]. Other than building debris, lead, glass, respirable asbestos fibers and other toxic particles from household materials are released as nanosized particles around the site of building demolition [103]. Cigarette smoke can lead to chronic respiratory illness, cardiovascular disease, pancreatic cancer [104], genetic alterations [105], middle ear disease and exacerbated asthma [104]. It is noteworthy that there is a chance to reverse the risks of myocardial infarction associated with inhaled NPs after smoking cessation [106]. The hazardous effect of demolition particles and their long-term effects towards humans are still unknown. However, respiratory symptoms such as a cough and bronchial hyperactivity were found among firefighters who participated in the rescue mission during World Trade Center on September 11, 2001 [107]. This indicates that extensive studies should be carried out amongst workers of demolition sites to identify the ill-effect of particles that are dissipated.

**Nanoparticles in biomedical and healthcare products:** NMs are incorporated in cosmetics and sunscreens as antioxidants [108] and antireflectants [109]. Mostly, NPs used for commercial applications are engineered NPs that are produced using physical [110], chemical [111] and biological methods [112]. As engineered NPs are attached to a firm surface, the risk of detachment and causing health issues is lessened [64]. Other than cosmetics, NPs have been extensively used in commercial products ranging from personal care products to paints [113]. Titanium oxide NPs larger than 100 nm are broadly utilized as a white pigment in cosmetic creams and sunscreens [114]. Similarly, Ag NPs have been used in diverse applications including air sanitizer sprays, wet wipes, food storage containers, shampoos, and toothpastes [115]. Several NPs are under research and evaluation of additives in personal care products. In spite of the emerging growth of products with different types of nanomaterials, their hazardous effects on humans are largely unknown. The extensive studies reported that Ag NPs demonstrated a size, morphology, and dosage-dependent higher cytotoxicity to humans and animals cells than asbestos [91,116–120]. The hazardous effects of other NPs present in consumer products are unknown and are still under research.

#### Naturally produced nanomaterials

Apart from incidental and engineered nanomaterials, nanoparticles and nanostructures are present in living organisms ranging from microorganisms, such as bacteria, algae and viruses, to complex organisms, such as plants, insects, birds, animals and humans. Recent developments in the equipment to visualize nanomaterials help in identifying the morphology of these naturally formed NMs, which will eventually lead to the better understanding of these organisms. The knowledge about the nanostructures present in microorganisms is important for the further use of these organisms for beneficial biomedical applications. Insects have nanostructures that are formed via an evolutionary process which helps them to survive in harsh living conditions. Plants also utilize the nutrients available in soil and water for their growth which leads to the accumulation of these biominerals in nano-form. Animals and small insects utilize nanostructures for their protection from predatory organisms as well as in their lightweight wings via nanowax coatings. Similarly, humans also possess organs that are primarily contructed by nanostructures, such as bones. Antibodies, enzymes and other secretions that are highly beneficial for the proper function of humans are found to be in nanometer size range. It can be also noted that the genetic material (DNA or RNA), which is important for the cell formation and function of all living cells, are nanostructures. This clearly shows that nanostructures are the basic foundation for all life forms on Earth. The following sections aim at listing the nanostructures that are present in living organisms.

**Nano-organisms:** Nanoscale organisms, commonly known as nano-organisms are found all around us and even inside our bodies. The category “nano-organisms” are naturally occuring nanomaterials that include a massive range of organisms, for example, nanobacteria, viruses as well as fungi, algae, and yeast that can produce nanoparticles in their bodies.

**Figure 4:**
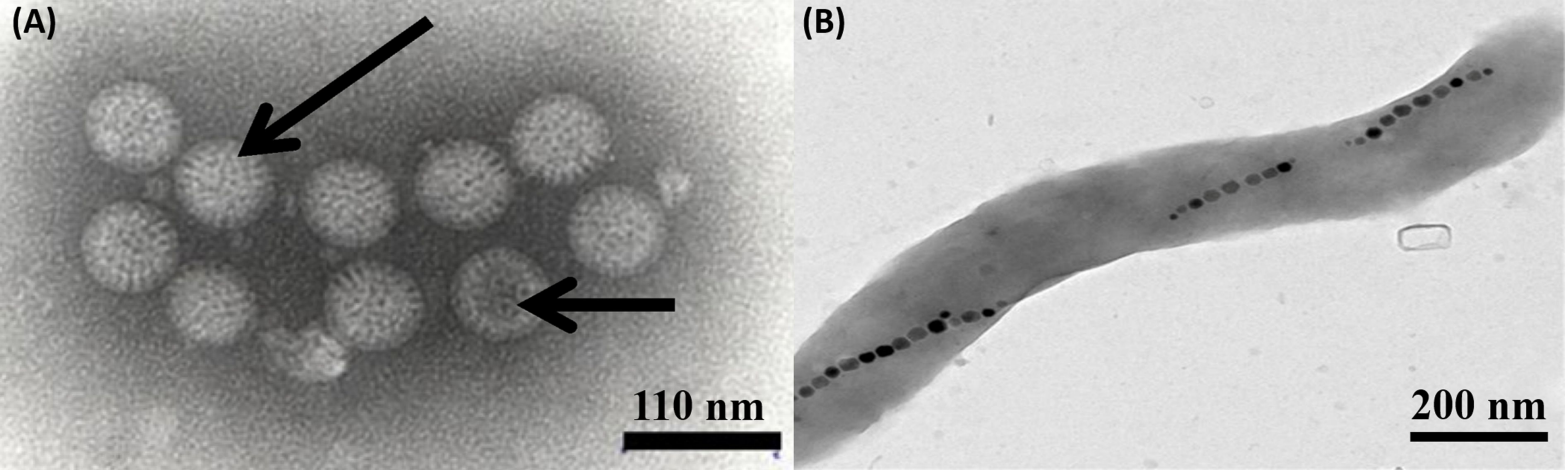
(A) Negatively stained rotavirus with complete (long arrow) and empty (short arrow) particles in swine feces [135], copyright Catroxo and Martins, 2015. (B) TEM image of a magnetotactic bacterium, reporduced with permission from [136], copyright 2014 Alphandéry.

**Viruses:** Viruses are the largest structurally characterized molecular assemblies known to date, which can be a non-living crystal and a living organism inside host cells. Generally, they are considered to be harmful as they cause disease in bacteria [121], plants [122], animals [123] and humans [124]. Advances in molecular biology have increased the possibility to genetically tailor viruses for use as catalysts and bio-scaffolds. Nanosize, monodispersity, distinct shapes, selective permeability to smaller molecules, composition controllability by genome manipulation, self-assembly and polyvalence, rapid growth, and stability towards pH and temperature [125–126], are properties that make viruses a unique category among NMs [127]. Viral NPs, as shown in Figure 4A, can be prepared from viruses by removing their genetic material and making them “nano-cargoes” for targeted drug delivery. Saunders et al. [128] described the development of viral NPs using RNA-removed cowpea mosaic virus through a proteolytic process. The nanocages or protein capsids were used to encapsulate drugs, genes, enzymes or proteins for targeted delivery with biocompatibility and bioavailability [128]. Recent research efforts have focused on using viral NPs as conjugation templates to produce novel nanostructures [129–130] and cages for compound encapsulation [131–132]. Plant viruses have been found to be nontoxic towards human cells at required dosages for effective administration of the drug load [133–134].

**Nanobacteria and nanobes:** Generally, bacteria will bind to soluble, toxic heavy metals and precipitate them to their surface, producing metal NPs. These are called as nanobacteria and are highly useful in the biosynthesis of low toxicity NPs [137]. *Pseudomonas stutzeri* A259 is the first bacteria to be used to produce Ag NPs [138]. Later, many metal NPs, such as gold [139–140], alloy NPs [141–142], nonmagnetic oxide NPs [143–147], and metal sulfide quantum dots such as CdS [148–149] and ZnS [150], were synthesized using different strains of bacteria. Other than bacteria, actinomycetes such as *Thermomonospora sp* and *Rhodococcus sp.* [151] are also used to produce NPs. This bacteria-mediated NP formation was found to be highly useful in a nanomedicine application as they were found to reduce potential cellular toxicity [152]. However, the major drawbacks of these NPs are that they require more time for synthesis, are difficult to filter, and produce a low yield of NPs, as compared to chemical synthesis [153].

Novel nano-organisms, called nanobes, are gaining interest among nanotechnology researchers as they are found during off-shore petroleum exploration on Triassic and Jurassic sandstones in Western Australia [154]. These nanobes contains 20–150 nm diameter individual cells that are composed of a carbon, oxygen, nitrogen, DNA, membrane-bound structure with dense cytoplasm and nuclear area as well as mineral compounds similar to actinomycetes and fungi. The uniqueness of nanobes is their size, which is well below the range considered to be viable for autonomous life on Earth, and that they were recently found in martian meteorite ALH84001 [155].

**Magnetotactic bacteria:** Magnetotactic bacteria are highly helpful to produce magnetic oxide NPs that possess unique properties such as superparamagnetism, high coercive force and microconfiguration, which can be utilized for biological separation and in biomedicine fields [152]. Generally, biocompatible magnetite (Fe_3_O_4_), iron oxide, iron sulfides and maghemite (Fe_2_O_3_) are synthesized using magnetotactic bacteria [156–157] that helps in targeted cancer treatment via magnetic hyperthermia, magnetic resonance imaging (MRI), DNA analysis and gene therapy [158]. Moreover, surface-distributed magnetic iron-sulfide particles [159], 12 nm magnetic octahedral NPs [160], modified iron NPs [161] and superparamagnetic NPs [162–163] were produced by using magnetotactic bacteria. Bacterial magnetic particle (BacMPs) [164] produced via bacterium are suggested to perform as a bio-needle in a compass and helps those bacteria to migrate under the impact of the Earth’s geomagnetic field along with oxygen gradients in aquatic environments, as shown in Figure 4B [165]. Morphologies such as vibrio, cocci, spirilla, rod-shape, ovoid and multicellular bacteria are found to possess unique characteristics in yielding NPs [164–167]. The NP formation mechanism is under extensive debate and revealing the mechanism will help in further improvement of the magnetotactic-bacteria-based NP synthesis in the future.

**Figure 5:**
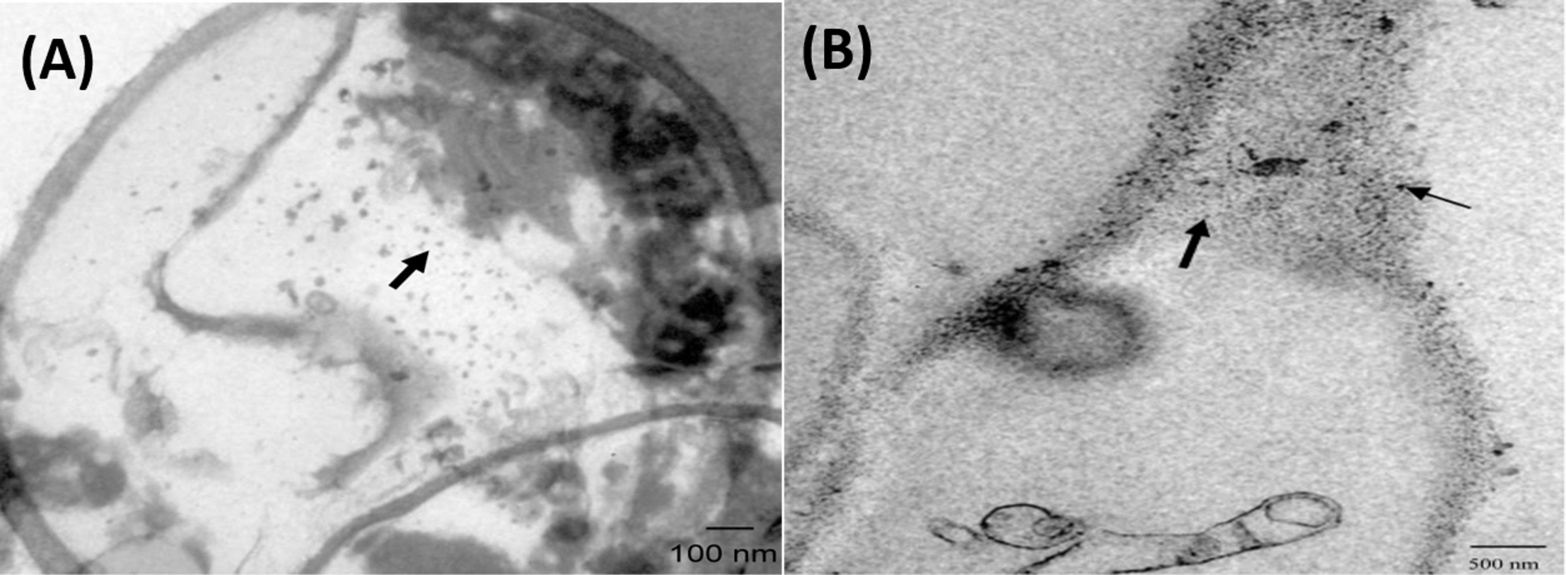
Nanoparticles synthesized intracellularly in algae and fungi. (A) TEM micrograph of *R. mucilaginosa* yeast section showing (arrow) intracellular localization of Cu NPs [185], copyright 2015, Salvadori et al. (B) TEM photomicrograph of dead *H. lixii* fungal biomass section showing extracellular (lighter arrow) and intracellular (darker arrow) nickel oxide NPs [186], copyright 2015, Salvadori et al.

**Algae, fungi, yeast and bacterial spores:** Algae such as *Chlorella vulgaris* supports the formation of Ag NPs [168], phytochelatin-coated CdS by *Phaeodactylum tricornutum* [169], and nanocomposite and nanoporous structures via coccoliths and diatoms [139]. Since very limited studies are available, the possible mechanisms for algae-mediated nanoparticle formation are still unidentified [170]. Similarly, fungi are utilized for the synthesis of NPs and the literature suggested that they are excellent candidates for metal and metal sulfide nanoparticle synthesis, as shown in Figure 5B [171]. Fungi contain a variety of enzymes and are simple to handle, which gives the possibility of synthezing NPs with various sizes and shapes. It is noted that *Fusarium oxysporum* and *Verticillium sp.* of fungi have been noted to aid in Au, Ag and Au–Ag alloy NP synthesis [141,172–173]. Enzymes in *Fusarium oxysporum* fungi also help in the synthesis of CdS quantum dots [174] and serve as a source of sulfate reductases [171,174] and also in the formation of zirconium particles [175]. Moreover, yeasts namely *Candida glabrata*, *Torulopsis sp.*, *Schizosaccharomyces pombe* and MKY3 (which is a yeast strain with tolerance of Ag) were also used in the synthesis of NPs such as CdS quantum dots [176–177], PbS nanocrystals [178] and Ag NPs [179], respectively, as shown in Figure 5A. Recently, it was found that the spores of bacteria such as *Bacillus anthracis* on the nanoscale can cause food contamination and contagious diseases [180]. Similarly, a list of autotrophic plants and heterotrophic microbes that help in the formation of Ag NPs along with possible nucleation mechanisms are presented in recent review articles [153,181–184]. This list assists in identifying the crucial factor that induces nanoparticle nucleation. This identification results in the preparation of nanometer-sized targeted drugs that can inhibit the growth of these harmful bacteria in its early stage.

#### Nanoparticles and nanostructures in plants

Wood is made of natural fibers that are considered as cellular hierarchical bio-composites. Natural fibers are composites of cellulosic-fibrils at the nanoscale level. The simplest form of nanometer-sized cellulosic-fibrils are 100–1000 nm long, containing both crystalline and amorphous segments. The unique strength and extreme performance properties of various natural fibers such as wood are attributed to their elementary hierarchical structure with nanofibrillar components [187]. The isolation of nanocellulose from natural sources is possible through nanotechnology, which requires combined methodologies including mechanical, chemical and other processes. The resulting cellulose nanofibers could have distinct morphologies such as a rod-like NPs (whiskers) or an entangled network (nanofibers) [188].

**Figure 6:**
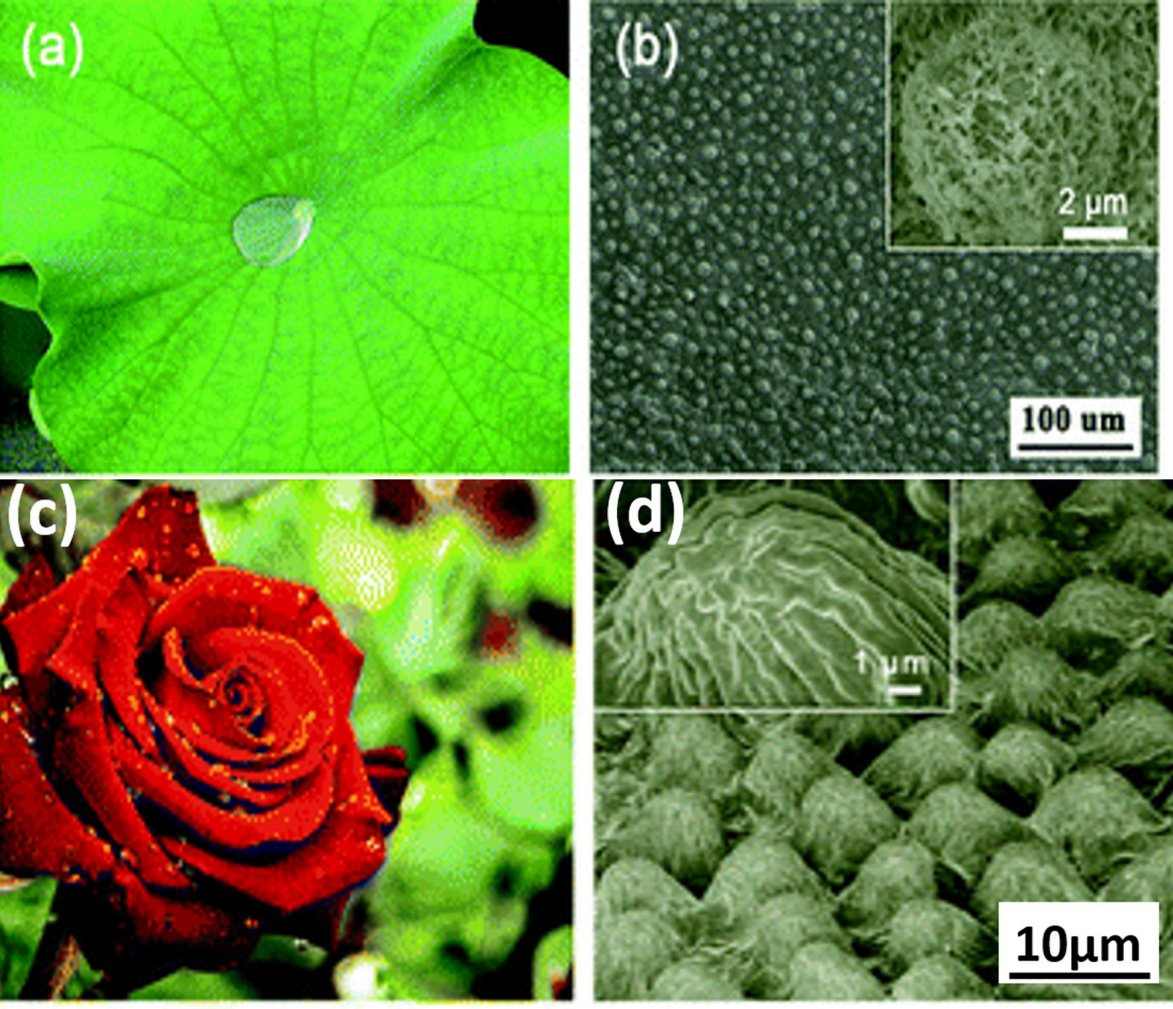
Photographs and the scanning electron microscope images of various bio-prototypes bearing superhydrophobic surfaces. (a) Photograph of a lotus leaf; (b) SEM image of the lotus leaf surface. The inset is a SEM image of a typical 5–9 µm micropapillae covering the surface with fine branch-like nanostructures [210], copyright 2002 Wiley-VCH. (c) Photograph of a red rose and (d) SEM image of a rose petal surface. The inset is a magnified SEM image of the microcapillary arrays [196,211], copyright 2008, American Chemical Society.

Plant surfaces, especially leaves, contain nanostructures that are used for numerous purposes such as insects sliding [189], mechanical stability [190], increased visible light and harmful UV reflection and radiation absorption respectively [191–192] as shown in Figure 6. The most famous nanostructure property in plants is the superhydrophobicity in lotus leaves that helps in self-cleaning and super-wettability of the leaves [193]. Many studies in the literature have suggested that stacks of nanostructures are responsible for the circular layer in plants and insects which allows them to float on water without sinking [194–195]. Based on these reports, many artificial superhydrophobic materials with self-cleaning ability have been manufactured [196] through electrodeposition, photolithography and colloidal systems [197–199] with unique morphology and roughness [200–201]. These superhydrophobic materials were useful in applications such as water treatment [202–203], wettability switchers [204–205], smart actuators [206], transparent coatings and electrodes [207–209].

#### Nanoparticles and nanostructures in insects

Insect wing membranes are comprised of building materials with 0.5 µm to 1 mm thickness [212]. Additionally, the insect wings are formed by a complex vein system which gives superior stability to the entire wing structure [213–215]. Long chain crystalline chitin polymer is the basic framework of insect wings that provides membrane support and allows for bearing forces on them during flight [216–217]. Resilin enhances the wing’s flexibility and is a unique component that is found in between the junctions of the vein and the wing [217–219]. The routine and longer colonization flights were supported by the vein system along with their weightless wing material [220–222]. Insect wing surfaces demonstrate a rough and highly ordered structure comprised of micro- and nanoscale properties to minimize their mass and protect them against wetting and pollutants. A methodical terminology to explain the structural properties of insect cuticles was developed and mentioned in a review by Byun et al. [223]. The review focused on describing the structures using SEM images and highlights distinct insect wing morphologies. Generally, the characteristics of wax crystals that exist on the wing surfaces are described by the terms “Setae”, “denticles” and “fractal”. The setae are needle or hair-like structures with a high aspect ratio; a denticle is structured with morphology ranging from smaller hemispherical to taller fractal; pillars are fine irregular nanoscale projections [223]. SEM images and photographs of various insect species and orders are provided. It is observed that wood termite (*Schedorhinotermes* sp.) and cicada (*Meimuna microdon*) wings are concealed by a denticle layer, while hornet (*Vespa* sp.) wings are covered by multiple setae. The water contact angles (WCA) are observed to be less than 150° for both the structures [224–226] and are not considered as superhydrophobic. Conversely, a WCA greater than 150° was exhibited by the wing of the grasshopper (*Acrida cinerea cinerea*), dragonfly (*Hemicordulia tau*) and butterfly species (*Papilio xuthus*) over their surface. The literature also show that species with sophisticated fractal and layered cuticle patterns possess superhydrophobic properties. These structural types are composed of the hierarchical structure which may be responsible for increasing the surface hydrophobicity [194]. Moreover, the colors of butterflies are attributed to their fine wing structure. Indeed, the literature reveals that they possess nanostructures in multilayers which act as diffraction gratings, induce interference, and consequently iridescence [227–228].

#### Nanoparticles and nanostructures in animals and birds

**Figure 7:**
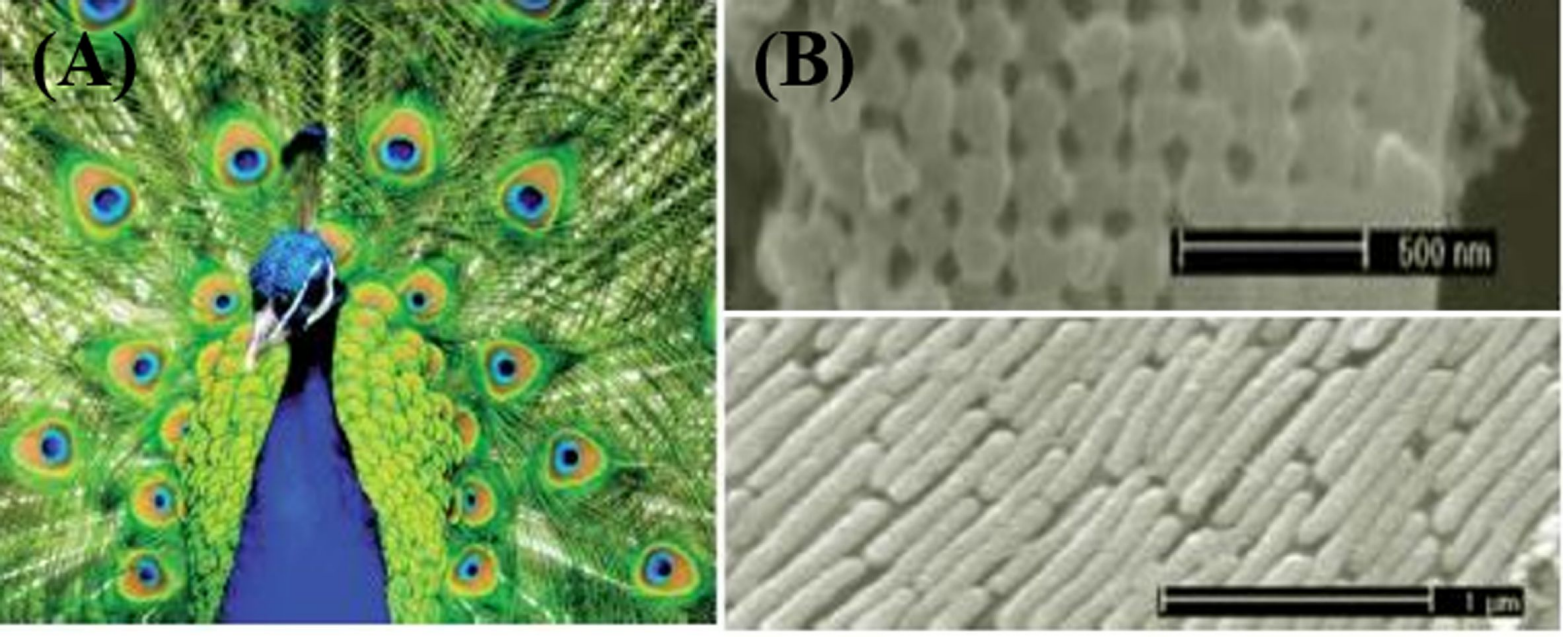
(A) Photograph of peacock feathers showing various colors and patterns. (B) Cross-sectional SEM images of the transverse (top) and longitudinal (bottom) sectionals of green barbule cortex [196], copyright 2012, Royal Society of Chemistry.

Animals (insects belonging to Kingdom Animalia) such as flies, spiders, and geckos with varying body weight can attach along ceilings and move along vertical walls. The interaction of their patterned surface structure with the substrate profile gives efficient ability and mechanism for attachment to the insect’s legs. An intense inverse scaling effect in these attachment devices are exposed via an extensive microscopic study. It has been shown that adhesion is ensured by sub-micrometric devices whereas flies and beetles rely on terminal setae that are of micrometer dimensions. The principle of contact mechanics, which shows that the adhesion leads to the splitting of contacts into finer subcontacts, helps to clearly explain the insect body weight to setae trend. The natural adhesive system uses this principle for their design and may be incorporated in future practical applications. Research on attachment and mechanism of insects walking on ceilings using their hairy attachment systems began 300 years ago and continues today. Electrostatic forces, sticking fluids, and microsuckers are the proposed reasons that explain the insect’s attachment mechanism [229]. Some of these theories have been rejected based on experimental data and combination of secretion-mediated capillary attractive forces and molecular interactions [230] or van der Waals interactions leads to adhesion [231]. This may be due to the production of secretory fluids in the contact area by some animals (insects) [232–234], whereas others do not (spiders, geckos) [235–236], which makes the basic force in the physical form contribute to their adhesion. In recent reports, the reason for adhesion of gecko setae is due to van der Waals interaction through strong evidence [237] and rejects the capillary adhesion mechanisms. It was predicted that application of contact mechanics may help in smaller setae array endings by releasing greater adhesive strength [237–239]. The beautiful color patterns of peacock feathers are also known to be due to the cross-sectional arrangement of their feather frills as shown in Figure 7 [196].

Mollusk shells consists of “nacre”, which is a hierarchical nanocomposite. Nacre is designed by alternating micrometer-sized and sub-micrometer CaCO_3_ aragonite platelets, which are separated by a thin layer of bio-macromolecular “glue”. Enhanced stiffness, impact resistance, strength, and toughness are some of the mechanical properties that enable using nacre’s unique design. The nacreous effect is caused by the thin layer of a rough surface with groovy nanostructures [240]. Other than the nacreous effect, gecko feet have the capability to walk on ceilings against gravity and even on wet or slippery surfaces. This property is linked to the nanometer-sized hair-like structures in their feet that are aligned in a series of a small ridges with a projection of 200 nm width in each hair. This increases the total surface area of gecko feet and leads to a van der Waals interaction mediated strong surface adhesion [241]. Similarly, the crystalline composite of CaCO_3_ crystals and protein that are aligned in a column and layers of calcite, forms the thin and strong eggshell. During the eggshell formation, the CaCO_3_ NPs begin as an amorphous mineral which is transformed by the c-type lectin proteins into ordered crystals. The crystal transformation is initiated by the attachment of proteins towards ACC NPs and later detach when the crystal continues to grow [242].

#### Nanoparticles and nanostructures in the human body

The human body consists of nanostructures without which normal function of the body is impossible. It is formed by nanostructures such as bones, enzymes, proteins, antibodies and DNA. A list of nanostructures that exist in the human body is presented in Table 1. Even some works categorize bone as a nanomaterial comprised of hierarchical inorganic nano-hydroxyapatite and organic collagen [243]. Additionally, micro-organisms such as viruses and bacteria are nanostructures that can cause diseases in humans.

**Table 1:** List of nanostructured particles associated with the human body.

Nanostructure	Size	Ref.

glucose	1 nm	[244]
DNA	2.2–2.6 nm	[245]
average size of protein (rubisco monomer)	3–6 nm	[246]
haemoglobin	6.5 nm	[244]
micelle	13 nm	[244]
ribosomes	25 nm	[247]
enzymes and antibodies	2–200 nm	[248]

#### Bone nanostructures

**Figure 8:**
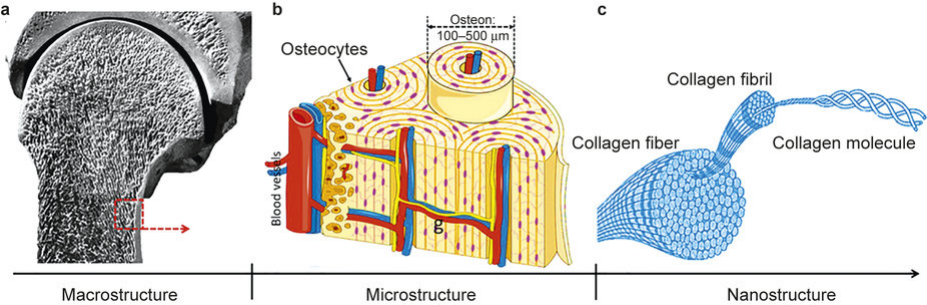
The macro- and microstructure of bone and its components with nanostructured materials employed in the regeneration of bone. (a) Macroscopic bone details with a dense cortical shell and cancellous bone with pores at both ends. (b) Repeating osteon units within cortical bone. (c) Collagen fibers (100–2000 nm) comprised of collagen fibrils [254], copyright 2015, Springer Nature.

The inimitable combination of natural bone with precise and carefully engineered interfaces and mechanical properties is due to their nanoscale to macroscopic architectural design and dimensions. The interaction of micro/nanoscale components with the extracellular matrix (ECM) within the stem cells includes influential stem cell behavior through sources of passive mechanical force. A wide structural protein spectrum and polysaccharides of different length scales with dominating nanometer-sized collagen fibrils strands of 35–60 nm diameter and a micrometer range length comprise the main building blocks of the ECM [249]. Bone is a multifaceted composite with numerous hierarchical levels as shown in Figure 8. The cortical bone with a compact shell and the spongiosa or trabecular bone with a porous core are the two important parts of bone tissue (Figure 8a). Repeating osteon units together forms cortical bone whereas an interconnecting trabeculae framework with bone marrow and free space helps to form cancellous bone. Likewise, calcium phosphate crystals and collagen fibers are specifically arranged to form the trabeculae and osteon units. The collagen molecules are periodically arranged with gaps of 47 and 60 nm to form collagen fibrils (Figure 8b) [250–251]. The gaps in collagen fibrils are embedded with hydroxyapatite (HA) crystals to increase the bone rigidity (Figure 8c) [252–253]. The hierarchical organization with nanometer to centimeter magnitude and structure of the ECM and cells determines the properties of bone tissues [254–255].

#### DNA nanostructures

DNA is the genetic blueprint repository of living organisms. It helps in the synthesis of protein, which is essential for the activities of living organisms [256]. Mono-phosphorylated deoxyribose sugar attached with nitrogenated aromatic nucleobase is called a nucleotide, and this is the basic structural unit of DNA. DNA possesses diverse sequence information storage mechanisms with 2.86 bits per linear nanometer density [257]. A-DNA, B-DNA, and Z-DNA are three types of DNA classification based on the base-paring between the strands. B-DNA is a right-handed double-helical DNA structure [258–259] whereas A-DNA is a comparatively short, more-compact, right-handed double-helical structure, and Z-DNA is a left-handed double-helical DNA formed with long polypurine stretches [260–261]. These DNAs are nanostructures in organisms and their interactions with other NMs play a major role in nanomaterial drug formulations. Thus, in recent years, research on artificial DNA nanostructures have escalated in the field of bionanotechnology.

A phosphate backbone with negative charge, nucleobases with metal chelates, and the hydrophobic core with aromatic rings are the chemical handles that are responsible for the formation of self-assembled nanostructures through interaction with inorganic NMs [257]. The formation of DNA-templated metal nanostructures is possible by localizing transition metal cations on DNA to act as precursors and chemical handles [262]. DNA nanostructures [263] and DNA attached to NPs [264] have been synthesized for various applications including nanobarcoding and DNA sensors [265]. Research in this area has advanced to include active self-reconfiguration of 1, 2 or 3-dimensional DNA-based nanoscale architectures for drug delivery, molecular electronics and logics [266–269]. Recent developments in DNA technologies such as Holliday junction elucidation and crossovers help in the virtual assemblage of any DNA structures through DNA origami. An extensive review on DNA origami, their functions and potential has been reported in [270]. They mentioned that NP-templated DNA and hybridization-based DNA are revolutionary particles that will create a positive impact on future biomedical fields.

#### Other nanostructures in the human body

Antibodies, enzymes, proteins and most organelles within cells are smaller than the micrometer-scale and are considered nanostructures. Recently, lipids, self-assembled peptides, and polysaccharides were also included in the list of nanostructures present in the human body [271–272]. These nanostructures are artificially manipulated for use in pharmaceutical industries. Nanozyme, which is an example of such nanostructures, is an engineered nanometer-scaled artificial enzyme [273]. The enzyme functions to mimic the general natural enzyme principles [274–275]. Cyclodextrins, porphyrins, supramolecules, polymers and biomolecules, which include antibodies, nucleic acids and proteins, have been widely investigated to imitate the structure and function of natural enzymes. Nanozymes are already under research for applications in biosensing, immunoassays, stem cell growth and environmental rehabilitation via pollutant removal [276]. As mentioned in the previous section, viral protein capsids are extensively under research investigation as self-assembling NPs. Aside from that, manipulation of natural proteins and antibodies with NPs [277–278] as well as individual proteins/antibodies [279–280] are gaining positive biomedical applications. It is believed that these biomolecular NPs will be highly beneficial for efficient biomolecule delivery and in therapies and diagnostics for complex diseases and genetic disorders.

### Challenges and risk assessment of nanomaterials

Recent articles and the frameworks reviewed in previous studies, outline the general properties of NMs regarding risk assessment. These properties are based on the essential characteristics of the NPs that are directly related to their synthesis methods [281]. The properties of NPs and their impact in inhibiting challenges and toxicity risks are summarized in Table 2.

**Table 2:** Summary of five basic nanomaterial properties and their potential risks and challenges.

Nanomaterial properties	Risk description

agglomeration or aggregation	Weakly bound (agglomeration) and fused particles are significant risk criteria as they lead to poor corrosion resistance, high solubility and phase change of NMs. This further leads to deterioration and the structure maintenance becomes challenging [282–283].
reactivity or charge	NPs can be charged either by functionalization or spontaneous degradative reactions. Chemical species and their charge-related critical functional groups will be a significant factor for specific functionality and bioavailability of NMs [284].
impurity	Inherently, NPs interact with impurities due to their high reactivity. Due to this reason, encapsulation becomes a prime necessity for solution-based NP synthesis (chemical route). In the encapsulation process, the reactive nano-entities are encapsulated by nonreactive species to provide stability to the NPs.
contaminant dissociation	The contamination of residual impurities in the NP is considered as a major risk factor. For example, sulfur impurities may present in iron oxide NPs depending on the precursor used for their production (FeCl_3_ or Fe_2_(SO_4_)_3_). Similarly, nickel, yttrium, or rubidium metal impurities may be present in the carbon nanotubes (CNTs) [285–286] that are adsorbed on the CNT surface.
size	Reactivity and agglomeration of NPs is mostly dependent on their particle size. It is well known that the process of agglomeration will happen at slower rates in smaller particles. After the synthesis of the NPs, it is impossible to retain their original size. Hence, encapsulation becomes highly inevitable in NP synthesis. The exceptional size-dependent chemistry of NPs is distinguished from classical colloid chemistry by categorizing NPs according to their particle size [284].
recycling and disposal	NMs are not bound to any hard-and-fast safe disposal policies. The experimental results of NP exposure are not available and their potential toxicity issues are still under question. Hence, the uncertainty of a nanomaterial’s effect is yet to be developed for permanent disposal and recycling policies.

#### Nanomaterial toxicity

Humans are exposed to NPs as they are produced by natural processes [64]. Production, use, disposal, and waste treatment of products containing nanoproducts are the prime reasons for the environmental release of nanoparticulates in the original or modified forms. Foreign substances are generally blocked by human skin, whereas organs susceptible to foreign substances include the lungs and gastrointestinal tract. NPs are comparable to viruses in size. For instance, the diameter of the human immunodeficiency virus (HIV) particle is on the order of 100 nm [64]. NPs that are inhaled can effortlessly reach the bloodstream and other sites in the human body including the liver, heart or blood cells. It is significant to mention that the toxicity of NPs depends on their origin. Many of them seem to be nontoxic and others have positive health effects [287].

The small size of NPs facilitates translocation of active chemical species from organismal barriers such as the skin, lung, body tissues and organs. Thus, irreversible oxidative stress, organelle damage, asthma, and cancer can be caused by NPs depending on their composition. The general acute toxic effects caused by exposure to NPs and nanostructured materials include reactive oxygen species generation, protein denaturation, mitochondrial disconcertion and perturbation of phagocytic functions. Uptake by the reticuloendothelial system, nucleus, neuronal tissue and the generation of neoantigens that causes possible organ enlargement and dysfunction are common chronic toxic effects of NPs.

Dimensionality, composition, morphology, agglomeration and uniformity are the general properties of NPs that are used to classify them. Similarly, nanostructured thin films or fixed nanoscale circuits within computer microprocessors and free NPs also possess vital differences which are easier for their applicational classification. There is no constraint for free NP movement, which makes them easier to spread throughout environmental and impose potential health risk via to human exposure. Conversely, proper handling of fixed NPs, where the nanostructured elements are attached to a large object, does not cause any health risk. Asbestos is a perfect example for this case where their primary states are safe. Later, the mining of asbestos leads to the production of nanoscale fibrous particles that are transformed into an airborne aerosol, carcinogenic and cause significant health hazard after absorbed in the lungs [64]. It is also noteworthy that the chemical composition and shape of the particle are the main factors contributing to nanoparticle toxicity, other than size and aging. In this context, many NPs are nontoxic, while others have reduced toxicity or may also have progressive health effects [64].

**Figure 9:**
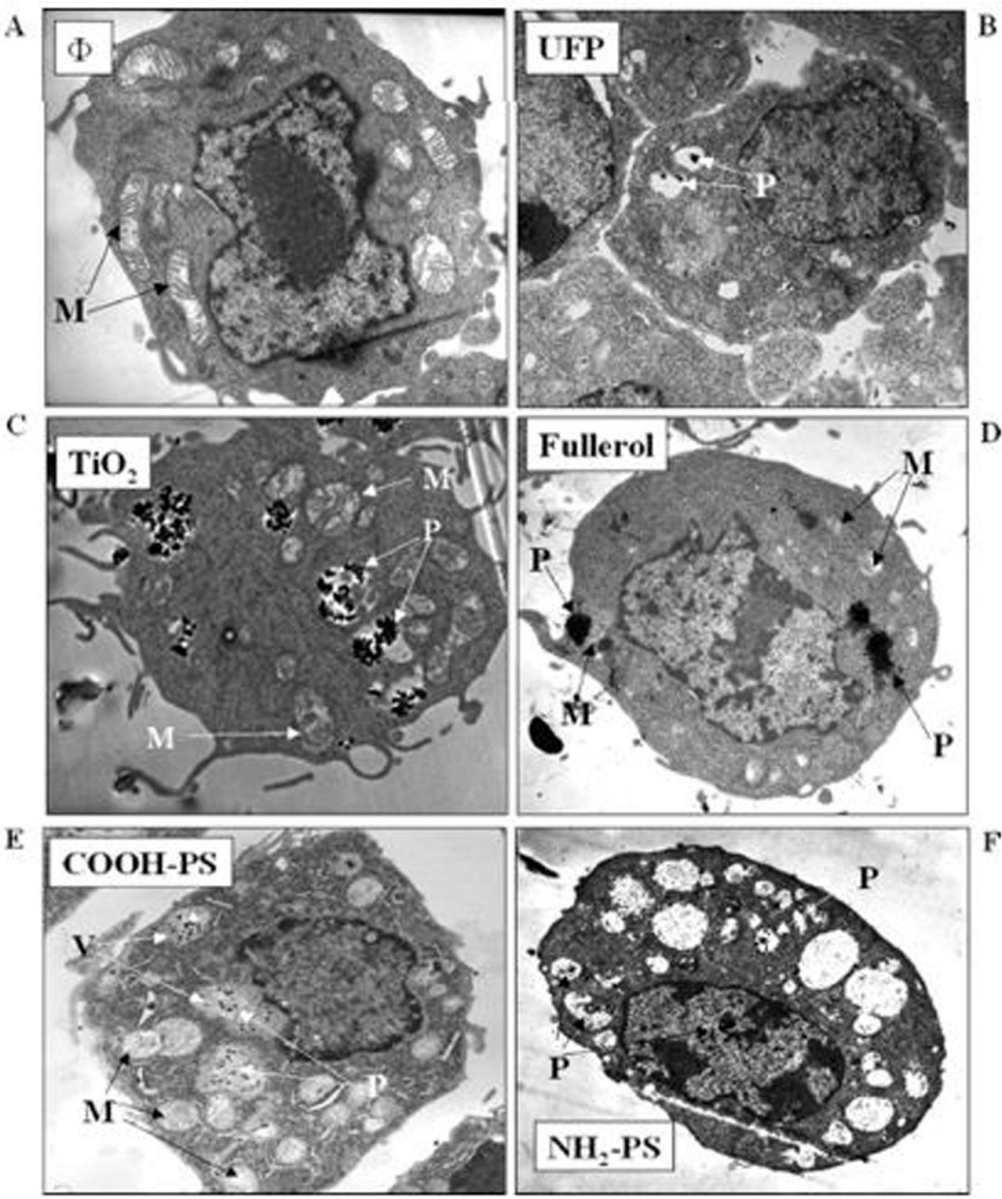
Electron microscope images show how NPs can penetrate and relocate to various sites inside a phagocytic cell line. (A) Untreated phagocytic cell line (RAW 264.7). Cells were treated with (B) ultrafine particles (<100 nm) (C) TiO_2_, (D) fullerol, (E) COOH–polystyrene nanospheres, and (F) NH_2_polystyrene nanospheres. NP exposure was conducted by treating the cells with 10 μg/mL NPs (<100 nm) for 16 h. Labels: M = mitochondria, P = particles [288], copyright 1969, Americal Chemical Society.

Foreign NPs lead to irreversible cell damage through oxidative stress or/and organelle injury with their cellular penetration and translocation ability [64]. Other than penetration, electrostatic charges, van der Waals forces, interfacial tension effects and steric interaction of NPs bind with cellular components and cause cell death [64] as shown in Figure 9. A wide variety of NPs can create reactive oxygen species and cause cellular damage via lipid peroxidation, protein alteration, DNA disruption, signaling function interference and gene transcription modulation [64]. The fate of oxidative products relies on the chemistry, shape, size and location of the NPs. Nanoparticles can relocate or distribute to various cellular sites such as the cytoplasm, components of cytoplasm and nucleus. NPs can harm cell organelles or DNA and cause cell mortality with their cellular localization effect.

According to toxicological data, the toxicity of NMs depends on various factors:

- Dose and exposure time effect. The number of NMs that penetrate the cells directly depend on the molar concentration of NPs in the adjacent medium multiplied by the exposure time [64].
- Aggregation and concentration effect. There are many contradictory reports on the toxicity of NPs at different concentrations. Increasing the NP concentration promotes aggregation. Most NP aggregates are micrometer in size, so that a significant quantity of aggregated NPs may not penetrate cells thereby losing their toxicity.
- Particle size effect. NPs show a size-dependent toxicity. Ag NPs with ≈10 nm diameter show a higher capacity to penetrate and disturb cellular systems of many organisms than Ag^+^ ions and Ag NPs of larger diameters (20–100 nm) [289].
- Particle shape effect. NPs exhibit shape-dependent toxicity, that is, different toxicity levels at different aspect ratios. For example, asbestos fibers of 10 µm length can cause lung cancer, shorter asbestos fibers (5–10 µm) can cause mesothelioma and 2 µm length fibers can cause asbestosis [290].
- Surface area effect. Typically, the toxicological effect of NPs increases with decreasing particle size and increasing surface area. It can also be noted that nano and microparticles with the same mass dose react differently with the human cells.
- Crystal structure effect. Based on the crystal structure, NPs may exhibit different cellular uptake, oxidative mechanisms and subcellular localization [288]. For example, the two crystalline polymorphs of TiO_2_ (rutile and anatase) show different toxicity. In the dark, rutile NPs (200 nm) lead to DNA damage via oxidation, while anatase NPs (200 nm) do not induce DNA damage in dark conditions [291].
- Surface functionalization effect. The surface properties of NPs have shown drastic effects relating to translocation and subsequent oxidation processes [292–293].
- Pre-exposure effect. The cellular phagocytic activity can be stimulated by shorter exposure time or the pre-exposure of lower NP concentrations [64]. This pre-exposure results in the adaptability of the human body against NPs to some degree [294].

#### Nanomaterial regulations

Nanomaterials possess characteristics such as high chemical bioactivity and reactivity, cellular as well as tissue and organ penetration ability, and greater bioavailability. These unique properties of NMs make them superior in biomedical applications. However, these merits are also avenues for potential toxicity. Thus, regulations via legislation, laws, and rules have been implemented by several government organizations to minimize or avoid risks associated with NMs [113]. However, there is no specific international regulation, no internationally agreed upon protocols or legal definitions for production, handling or labeling, testing toxicity and evaluating the environmental impact of NPs.

Medical standards related to ethics, environmental safety, and medical governance have been modifed to cover the introduction of NMs into the biomedical field [295–296]. Currently, the USA and the European Union (EU) have strong regulatory bodies and guideline legislation to control the potential risks of NMs. The European Commission has developed several pieces of EU legislation and technical guidance, with specific references to NMs. This legislation has been employed inside EU countries to ensure conformity across legislative areas and to guarantee that a NM in one sector will also be treated as such when it is used in another sector. According to the European Commission the term nanomaterial means "a natural, incidental or manufactured material containing particles, in an unbound state or as an aggregate or as an agglomerate, and where for 50% or more of the particles in the number size distribution, one or more external dimensions is in the size range of 1 nm to 100 nm". As the specifications of the materials and products meet the substance definitions of the European chemical agency (REACH) and the European Classification and Labelling of Chemicals (CLP), the provisions in these regulations apply [297]. In addition, the EU has formed the Scientific Committee on Emerging and Newly Identified Health Risks (SCENIHR), to estimate risks associated with NMs [298]. In 2013, EU cosmetics regulation 1223/2009 was replaced by Directive 76/768/EEC. The regulation defines the term nanomaterial as “an insoluble or bio-persistent and intentionally manufactured material with one or more external dimensions, or an internal structure in the range of 1 to 100 nm which includes man-made fullerene, single-walled carbon nanotubes, and graphene flakes”. It can be noted that cosmetics face regulations and moderations from USFDA’s Federal Food, Drug, and Cosmetic Act (FFDCA), Personal Care Products Council (PCPC), Voluntary Cosmetic Registration Progam (VCRP), EU cosmetics product notification portal (CPNP), REACH, Scientific Committee on Consumer Safety (SCCS) and International Cooperation on Cosmetic Regulation (ICCR). These regulations from the US and EU, as well as other countries such as Japan and Canada, reveal that nanotoxicity via cosmetics are of major concern for both scientific policymakers and industries producing consumer products [299–300].

In the US, regulatory agencies such as the Food and Drug Administration (FDA), the United States Environmental Protection Agency (USEPA) and the Institute for Food and Agricultural Standards (IFAS) have initiated protocols to deal with the possible risks of NMs and nanoproducts. Since 2006, the FDA has been working on identifying sources of NMs, estimating the environmental impact of NMs and their risks on people, animals and plants, and how these risks could be avoided or mitigated [301].

The European Medicines Agency (EMEA) and United States Food and Drug Administration (USFDA) help in regulating the medical usage of hazardous NMs. Apart from this, a book entitled “Principles for the Oversight of Nanotechnologies and Nanomaterials” was published by a coalition of US domestic and international advocacy groups and was endorsed by 70 groups on six continents. This article demands for a strong and comprehensive oversight of products generated from NMs. This encompasses a precautionary foundation for specific nanomaterial regulations, health, and safety of the public and workers, transparency, public participation, environmental protection, as well as the inclusion of broader impacts and manufacturer liability [302]. Similarly, the Nanomaterials Policy Recommendations report covers ways to avoid or reduce the risk of NMs in food-related industries. This report also advises companies to adopt a detailed public policy for NMs usage, publish safety analyses of NMs, issue supplier standards, label NPs below 500 nm and adopt a hazard control approach to prevent exposure to NPs [303]. Organic suppliers including the UK Soil Association [304], the Biological Farmers of Australia [305] and the Canada General Standards Board [306] have already banned the use of engineered NPs in food. Researchers and manufacturers should be educated on the regulatory laws and legislations prior to nanomaterial production to avoid these types of bans against NMs. It is currently agreed that NMs are not intrinsically hazardous per se and many of them seem to be nontoxic, while others have beneficial health effects. However, the risk assessment in the future will determine whether the NMs and their products are hazardous or any further actions are needed.

## Conclusion

The toxicity profiling of NMs is a highly demanded research area worldwide in recent times. Natural NMs have been present in the ecosystem for years, and they possess some mechanisms to cause less harmful effects among living organisms. However, research advancements have found some acute toxic effects of nanosized particles in living systems. From this review article, it can be noted that NMs from anthropogenic activities and engineered NMs in consumer products are able to cause toxic effects in living creatures. Additionally, emerging NPs, such as viral NPs and nanozymes, should be subjected to rigorous cytotoxicity tests to establish benign mechanisms of application and dosage levels. In order to minimize or avoid the potential hazards of engineered NMs in consumer products, regulations and laws have been implemented in many countries. Extensive research in the field of nanotoxicology and strict laws by government agencies are essential to identify and avoid toxic NPs.
